# Mental Health Consequences of Adversity in Australia: National Bushfires Associated With Increased Depressive Symptoms, While COVID-19 Pandemic Associated With Increased Symptoms of Anxiety

**DOI:** 10.3389/fpsyg.2021.635158

**Published:** 2021-05-19

**Authors:** Hussain-Abdulah Arjmand, Elizabeth Seabrook, David Bakker, Nikki Rickard

**Affiliations:** ^1^University of Melbourne, Melbourne, VIC, Australia; ^2^Centre for Mental Health, Swinburne University of Technology, Hawthorn, VIC, Australia; ^3^Cognitive Behaviour Therapy Research Unit, Institute for Social Neuroscience, Heidelberg, BW, Australia; ^4^School of Psychological Sciences, Monash University, Melbourne, VIC, Australia; ^5^Centre for Positive Psychology, Graduate School of Education, University of Melbourne, Melbourne, VIC, Australia

**Keywords:** COVID-19, depression, anxiety, public mental health, well-being, disasters, experience sampling methodology, smartphone

## Abstract

High quality monitoring of mental health and well-being over an extended period is essential to understand how communities respond to the COVID-19 pandemic and how to best tailor interventions. Multiple community threats may also have cumulative impact on mental health, so examination across several contexts is important. The objective of this study is to report on changes in mental health and well-being in response to the Australian bushfires and COVID-19 pandemic. This study utilized an Experience-Sampling-Method (ESM), using the smartphone-based mood monitoring application, MoodPrism. Participants were prompted once a day to complete a brief survey inquiring about symptoms of depression and anxiety, and several well-being indices, including arousal, emotional valence, self-esteem, motivation, social connectedness, meaning and purpose, and control. Participants were *N* = 755 Australians (aged 13 years and above) who downloaded and used MoodPrism, between 2018 and 2020. Results showed that anxiety symptoms significantly increased during the COVID-19 pandemic, but not during the bushfires. This may be explained by concurrent feelings of social connectedness maintained during the bushfires but not during the pandemic. In contrast, depressive symptoms increased significantly during the bushfires, which maintained during the pandemic. Most indices of well-being decreased significantly during the bushfires, and further again during the pandemic. Study findings highlight the unique responses to the bushfire and COVID-19 crises, revealing specific areas of resilience and vulnerability. Such information can help inform the development of public health interventions or individual clinical treatment, to improve treatment approaches and preparedness for potential future community disasters.

## Introduction

In a recent position paper, Holmes et al. (2020) urged prioritization of research which monitors and reports on mental health and emotional well-being in response to COVID-19. Survey research has shown that mental health and well-being significantly declined during the COVID-19 pandemic. Fisher et al. (2020) surveyed 13,829 Australians during the first month of COVID-19 restrictions. They found that symptoms of depression and anxiety were significantly higher than reported in normative data, with over 45% of the sample reporting at least mild symptoms of depression or anxiety. In addition to this, preliminary survey data from 2,297 Australians sampled across 1 week in April showed that there had been a 22% increase in boredom, 15% increase in stress, 14% increase in loneliness, coupled with a 15% decrease in optimism and 14% drop in happiness compared to respondents self-reported ratings of their mental health in general (Liddy et al., 2020).

Australia’s entry into the COVID-19 pandemic was distinct from the rest of the world. Bushfires had impacted the eastern coast continuously for 19 weeks, with over 18.6 million hectares of land burnt, 28 fatalities, and over 1 billion livestock and wildlife killed. In addition to the immediate threat of fire to communities, the air quality across the eastern seaboard deteriorated, hitting hazardous levels for several days. The damage caused by particulate matter on respiratory health was estimated to be responsible for 417 additional deaths, 1,124 hospitalisations, and 1,305 asthma cases that required attendance at emergency departments (Borchers Arriagada et al., 2020). The mental health and well-being impact of the bushfires was evident in early January, with increases in anxiety or worry strongly associated with indirect exposure to the bushfire threat (Biddle et al., 2020). Recent work has suggested that exposure to multiple community-level disasters has an additive detrimental impact on mental health (Harville et al., 2018). Taken together, the eastern states of Australia were already in a state of emergency and had been responding to public health concerns on a mass scale for a sustained period. The impact of COVID-19 on Australians must, therefore, also be considered in the context of this previous public health threat, rather than in isolation.

While cross-sectional insights have provided a snapshot of the acute impact of the COVID-19 pandemic on Australians mental health, little is currently known about the mental health impact emerging from the consecutive community threats that presented across 2019/2020 with reference to pre-disaster data. For example, is any increase in expression of depressive or anxiety symptoms attributed to COVID-19 greater than that experienced during other disasters, and how does it compare to regular yearly fluctuations that occur across seasons? To better capture the incidence of mental health issues in the general community, Holmes et al. (2020) recommend going beyond traditional health department data sets, and utilizing ecological momentary assessment (EMA) methodologies, also known as Experience-Sampling-Methods (ESM), to provide a dynamic picture of health and behavior. These assessment protocols allow sampling of participants’ experiences in real-time for extended periods, and overcome retrospective biases of survey research (such as mood congruency and recency effects) by accessing emotional reports as they occur. Monitoring population mental health and well-being is critical to understanding how communities respond to emergencies, their trajectories of recovery, and to better tailor opportunities for support and intervention. Smartphone dissemination of EMA intensive surveying may provide the high temporal resolution required during a pandemic and a useful avenue for ongoing public mental health surveillance. Inclusion of positive functioning indices has also been recommended to provide insight into resilience during the pandemic (Holmes et al., 2020) and to acknowledge the interactions between mental health and well-being (Australian Government, 2020).

Two EMA studies have examined the impact of the coronavirus pandemic on mental health focusing on the initial weeks of the outbreak. Fried et al. (2020) examined the short-term dynamics of mental health, social connectedness, and COVID-19 related concerns of Dutch university students over 2 weeks in late March. During this time, they found minimal change in mental health symptom severity and social connection. Due to the brief timeframe examined and absence of pre-pandemic comparison period, it is unclear if the stability of mental health indices was unique to the coronavirus period. In contrast, Huckins et al. (2020) reported on the mental health of 217 university students enrolled in the US-based longitudinal EMA study, StudentLife. Across a 10 week period from January 6, 2020, they found that symptoms of depression and anxiety were greater when compared to previous academic terms. Further, a specific spike in symptom severity was observed in the final 3 weeks of observation that coincided with home isolation restrictions.

Taken together, existing research highlights three important considerations: (a) the importance of collecting pre-pandemic data comparison data to enable some level of causal inference for the subjective experiences of community disasters, (b) the need for longer term observation to elucidate the evolution of mental health effects of Australians across multiple state emergencies, and (c) the need for assessing positive indices of mental health. In this paper, we report on experience-sampling data collected in Australian smartphone users, highlighting clinically relevant changes in mental health and well-being outcomes across three key time periods: baseline (normal public health); bushfires; and COVID-19 pandemic. Using intensively sampled longitudinal data collected by the MoodPrism app, we aim to provide a moving snapshot of the mental health and well-being impacts of the 2019-2020 Bushfire crisis and the coronavirus pandemic among Australian app users.

## Materials and Methods

### Participants

Data were collected from 1,509 participants who downloaded the experience-sampling application (MoodPrism; Rickard et al., 2016). A subset of 775 participants who used the app for at least 4 days (thus completing a minimum of four individual daily reports) was retained for data analysis. Participants were aged 13 years or above, and approximately 89% of the sample was located on the East Coast of Australia (where the 2019–2020 bushfires impacted). Although no other demographics were available from this sample, the app was available *via* both Android and Apple devices, which provides good representation of the greater population, as smartphone penetration in the Australian population is estimated to be 91% (Deloitte, 2019). Due to the open nature of recruitment, however, self-selection into the study may have enabled some bias in the sample composition. For example, younger demographics more familiar with smartphone technology could be more inclined to engage with the MoodPrism app. Similarly, individuals already experiencing symptoms of depression or anxiety may more readily download the app; in previous research utilizing smartphone-based mental health apps, individuals with likely diagnosable depression and anxiety have been well-represented in study samples (Bakker et al., 2018). It should also be noted that those with limited access to technology (low digital inclusion) are unlikely to be represented in this sample, a group that may be disproportionately impacted by the primary and secondary stressors associated with both the bushfires and coronavirus pandemic.

### Materials

Experience-sampling data were collected using the MoodPrism smartphone application (app). MoodPrism is an app available for download for Android and Apple devices.^1^ The app was designed in an ESM framework to monitor mental health and emotional well-being in real time by prompting users to complete a short survey daily (i.e., one survey per day), or Experience-Sampling-Report (ESR; Rickard et al., 2016).

Each ESR presented 12 items, rated along a 7-point scale. The first three items were semantic differentials with the anchor labels passive-alert (arousal), unpleasant-pleasant (emotional valence), and helpless-in control (personal control). Remaining items were Likert-type scales, with the anchor labels ranging from “Not at all” to “Extremely.” This included the 4-item Patient Health Questionnaire assessing symptoms of anxiety and depression (PHQ-4; Kroenke et al., 2009). The final five items were drawn from the positive psychology PERMA framework (14), and related to social connection, motivation, meaning and purpose, self-esteem, and a sense of achievement.

### Procedure

Participants were invited to download the app voluntarily as a personal mental health support tool from GooglePlay or the iOS App Store, and complete daily reports for a duration of their own choosing. Explanatory statements and consent forms were presented electronically through the app. If the reported date of birth was less than 18 years, an additional consent form for minors was presented, with checks for parental consent. Participants provided consent electronically by pressing an on-screen button indicating that they have read the explanatory statement and consent forms and agree to participate. Participants were also informed of incentives built into the research design, which included additional feedback on positive and negative mood functioning (unlocked after 1 and 2 weeks, respectively), and gaining entries into a prize draw for two cash vouchers ($50AUD).

All data collected from the Australian public from November 02, 2018 to June 15, 2020 were analyzed. The three periods of interest were defined as baseline (dates prior to August 31, 2019), bushfires (dates between September 01, 2019 and March 12, 2020), and COVID-19 pandemic (dates beyond March 13, 2020). Ethical approval was obtained from the Monash University Human Research Ethics Committee (Approval #19431).

### Data Analysis Plan

To examine changes in mental health and well-being across the three primary time periods, multilevel modeling (MLM) was utilized due to the nested structure of collected data. MLM accounts for the dependence of observations within participants, simultaneously estimates within- and between-person effects, while handling varying time intervals between entries and missing data (Bosker and Snijders, 1999; Krull and MacKinnon, 2001). In the current study, multiple daily ESRs (Level 1) were nested within individual participants (Level 2).

A model building procedure was adopted to conduct MLM analyses for each ESR item. For each model, the suitability for MLM was confirmed with a likelihood ratio test comparing the goodness-of-fit between an intercept-only model and a random intercept-only model. Random-intercept MLMs were then conducted for all variables with dummy-variable fixed-effects coded for each time period (baseline, bushfires, and COVID-19). The first MLM utilized the baseline period as the comparator to assess baseline vs. bushfire and baseline vs. COVID-19 differences, while a second MLM utilized the bushfires period as the comparator to assess bushfire-vs-COVID-19 differences. Partially standardized coefficients were produced from these models to provide a comparable estimate of the magnitude of effects across outcomes (Lorah, 2018).

To assist interpretation of the results, supplementary analyses were conducted exploring additional comparisons with, and between, equivalent time periods from the previous year to explore the feasibility of alternative interpretations (e.g., seasonal effects; see Supplement A).

## Results

Data preparation was conducted using SPSS version 25.0 (SPSS INC., 2013), and MLMs were performed using program R (R Cote Team, 2017). A total of 14,375 ESRs were completed during the data collection period. This comprised 4,923 reports during the baseline period, 5,034 reports during the bushfires period, and 4,418 reports during the COVID-19 period. The mean number of reports completed by each participant was 18.67 surveys (*SD* = 30.87, *range* = 4–357). Likelihood ratio tests for individual outcomes revealed significant improvements in model fit for random-intercept only models over intercept-only models (*p* < 0.0001 for all outcomes), confirming suitability of MLMs for each ESR item.

Table 1 presents the partially standardized coefficients from the MLM analyses comparing the three time periods.

**Table 1:** Partially standardized coefficients (standard errors) for individual multilevel models (MLMs) comparing average outcome scores across the baseline, bushfire, and COVID-19 time periods.

Outcome	Time period comparisons		
	Baseline^†^ vs. Bushfires	Bushfires^†^ vs. COVID-19	Baseline^†^ vs. COVID-19
Social connectedness	−0.01 (0.03)	−0.32 (0.03)^∗∗∗^	−0.34 (0.04)^∗∗∗^
Meaning and purpose	−0.11 (0.03)^∗∗∗^	−0.19 (0.03)^∗∗∗^	−0.30 (0.04)^∗∗∗^
Personal control	−0.08 (0.03)^∗^	−0.11 (0.03)^∗∗∗^	−0.19 (0.04)^∗∗∗^
Arousal	−0.02 (0.03)	−0.10 (0.03)^∗∗∗^	−0.12 (0.04)^∗∗∗^
Sense of achievement	−0.12 (0.03)^∗∗∗^	−0.10 (0.03)^∗∗∗^	−0.22 (0.04)^∗∗∗^
Anxiety symptoms	−0.02 (0.03)	0.09 (0.03)^∗∗^	0.08 (0.04)^∗^
Motivation	−0.09 (0.03)^∗∗^	−0.08 (0.03)^∗∗^	−0.17 (0.04)^∗∗∗^
Self-esteem	−0.08 (0.03)^∗^	−0.08 (0.03)^∗∗^	−0.16 (0.04)^∗∗∗^
Emotional valence	−0.06 (0.03)	−0.07 (0.03)^∗^	−0.12 (0.04)^∗∗^
Depressive symptoms	0.07 (0.03)^∗^	0.04 (0.03)	0.11 (0.04)^∗∗^

∗ *p* < 0.05;

∗∗ *p* < 0.01;

∗∗∗ *p* < 0.001.

† Comparator group.

**Figure 1:**
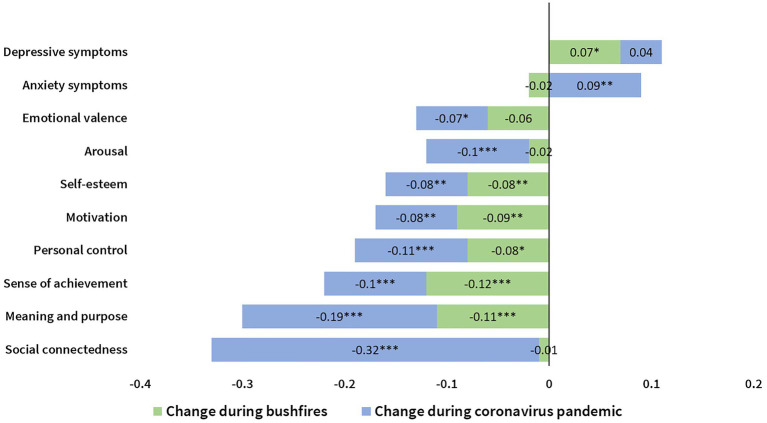
Visual representation of effect sizes for all measures in response to Bushfires (green bars) and COVID-19 pandemic (blue bars) relative to the baseline period.

These effects are presented visually in Figure 1.

Results showed several significant effects demonstrating standardized, average level change on mental health and well-being outcomes across the three time periods. First, a significant increase in anxiety symptoms, and a significant decrease in social connectedness, emotional valence, and arousal, was observed during COVID-19 when compared to the baseline or bushfire periods. In contrast, no significant change in these outcomes was observed when comparing the baseline period to the bushfire period.

Second, a significant increase in depressive symptoms was observed during both the bushfire and COVID-19 periods when each period was compared to baseline. However, no significant change in depressive symptoms was observed when comparing the bushfire and COVID-19 periods.

Third, significant cumulative decreases across all three time periods were observed for personal control, motivation, meaning and purpose, self-esteem, and sense of achievement with scores significantly lower during the bushfires period compared to the baseline period, and then significantly lower again in the COVID-19 period as compared to the bushfire period.

## Discussion

The current study investigated changes in day-to-day mental health and well-being of Australians as they experienced the 2019/2020 Australian bushfire and COVID-19 public health disasters. Several patterns of change in mental health and well-being differentiated these crises. Among 12 ESR variables monitored regularly by the smartphone app, MoodPrism, the two clearest indicators of population mental health and well-being are symptoms of depression and anxiety. Anxiety symptoms were not significantly impacted by the bushfires, but increased significantly with the onset of COVID-19. In contrast, depressive symptoms increased significantly during the bushfires, and remained elevated with the onset of COVID-19. Corresponding significant (and cumulative) decreases were also observed across several indices of positive functioning, elucidating the broad spectrum impacts of public health disasters on mental health and well-being.

The impact of COVID-19 on mental health reported in the current study is consistent with concurrent research reporting elevated levels of depression and anxiety during the early stages of COVID-19, compared to pre-pandemic data (Fisher et al., 2020; Huckins et al., 2020). The current study provides further insight into the evolution of mental health outcomes in response to the consecutive bushfire and pandemic community threats. Anxiety symptoms appeared to maintain at levels similar to those as during the baseline reference period despite a severe and protracted bushfire season; but then significantly increased in response to the COVID-19 pandemic and its associated restrictions. While this contrasts with the findings in relation to bushfires reported previously (Biddle et al., 2020), this indicates possible resilience to feelings of anxiety in response to bushfires present in Australian communities borne from past experiences with drought and bushfires. Such resilience may be generated through a sense of communal support community “togetherness,” or “mateship” deeply ingrained within Australian cultural values (Wilson et al., 2020). Supporting this interpretation, feelings of social connectedness also remained intact during the bushfire period (though did not buffer against symptoms of depression, discussed below). In contrast, the unique challenges and restrictions presented by COVID-19 deprived Australians of the ability to marshal or facilitate social support in conventional ways. This may have suppressed key strategies of community and individual resilience typically utilized in response to disasters (Emergency Management Victoria, 2020). The novelty of the threat posed by COVID-19 to Australian communities, who usually enjoy comparatively high standards of health, public safety, and public freedom, may have, therefore, rendered the population ill-equipped to effectively cope with feelings of stress and anxiety, as compared to more familiar disasters experienced historically.

Depressive symptoms were also increased in the COVID-19 period when compared to baseline. In contrast with anxiety symptoms, which remained at baseline period levels during the bushfires, depressive symptoms were significantly elevated and this remained with the onset of COVID-19 and transition into isolation restrictions. Consistent with cognitive models of depression, increased depressive symptoms during the bushfire period might be explained by feelings of inadequate personal control (Beck, 2002), or an inability to influence final outcomes, as most Australians could not mitigate the threats of the bushfires through action. Indeed, grief and sadness have been previously reported by Australians in responses to the environmental damage caused by bushfires (Block et al., 2019). While depressive symptoms did not increase significantly into the COVID-19 period, they remained elevated, which in combination with the increase in anxiety symptoms indicate a level of sustained psychological distress in the community.

Indices of positive functioning (well-being) demonstrated the greatest change from baseline and across disasters. The reduction in social connectedness was the largest effect of COVID-19 reported in this study, which is understandable given social distancing was an immediate public health response to the COVID-19, and a challenge that most had not previously experienced. Social support is widely regarded as protective against stress and anxiety (Ozbay et al., 2007), so reductions in social connectedness observed may partly explain increases in anxiety symptoms reported. As the pandemic has progressed, communities have adopted new ways of connecting through social media and other online platforms to maintain social contact. Further monitoring of social connection and anxiety levels during the subsequent infection waves would be of interest to assess whether these connection strategies alleviate anxiety levels (Seabrook et al., 2016), and should therefore be prioritized as a focus of clinical or public health interventions.

A reduction in meaning and purpose was the second largest effect observed in this study, which may point to the social, occupational, financial, and emotional impact of the COVID-19 restrictions. Large decreases in meaning and purpose were also observed during the bushfires, a target for clinical intervention following the disruptive trauma of bushfire disasters (Bateman, 2010). Meaning and purpose are recognized as key components of well-being and positive mental health functioning (Seligman, 2018) and have been found to offer individuals resilience during challenging times or traumatic events (Schaefer et al., 2013). As the impact of COVID-19 will continue to be felt into the future, cognitive-behavioral interventions that serve to improve perceived social connectedness, meaning, and purpose may have significant benefits on the mental health and well-being of the population. Interventions that improve perception of self-esteem, control, motivation, and achievement are also indicated as beneficial.

It is notable that many of the effect sizes reported are small relative to those found in clinical mental health research. While caution is, therefore, advised when drawing conclusions, small effects are not unexpected given the naturalistically sampled population and may still be relevant in addressing public health concerns. Another consideration is that study outcomes may be related to regular, yearly variations in mental health and well-being. Both observed (e.g., seasonal variation) or unobserved events may naturally occur concurrently with the dates defining each time period and may better explain changes observed across time periods. Supplementary analyses examined this possibility by examining whether the changes observed across the bushfire and COVID-19 time periods also occurred in the equivalent time periods of the previous year. These analyses revealed no such changes in the previous year, and also showed that the current bushfire and COVID-19 periods were significantly worse across most mental health outcomes than their respective equivalent time period in the previous year. These findings indicate that the source of change in outcomes observed in this study were unlikely due to any seasonal variations in mental health and well-being, and provide confidence that bushfire and COVID-19 community disasters were more probable causes. Finally, it is important to acknowledge potential self-selection biases in the study sample, as the “smartphone,” “mental health,” and “app” features of the study might have attracted particular groups of participants. This may include younger populations who are more experienced and familiar with smartphone technology, or individuals already experiencing existing symptoms of depression and anxiety (e.g., Bakker et al., 2018). While not confirmed in the present study, this possibility would limit the generalizability of study findings to such samples, and indicate that normative mental health responses to consecutive community disasters could differ from those presented hitherto.

Notwithstanding these limitations, this study demonstrates the impact of two distinct and consecutive community disasters on Australian mental health. Importantly, effects reported here are derived from real-time monitoring of day-to-day experience, and reflect changes in both mental health and well-being. This provides insight into an Australian profile of mental health response, highlighting specific areas of resilience and vulnerability. Such information can be used to inform the development of pre-emptive public health interventions or individual clinical treatment, to improve current treatment approaches and preparedness for potential future community disasters. By tailoring interventions to strengthen key areas of vulnerability, and support important mechanisms underlying resilience, individual capacities to cope under adverse circumstances may be improved, and the mental health consequences of community disasters may be more effectively managed.

## Data Availability Statement

The raw data supporting the conclusions of this article will be made available by the authors, without undue reservation.

## Ethics Statement

The studies involving human participants were reviewed and approved by Monash University Human Research Ethics Committee. Written informed consent to participate in this study was provided by the participants’ legal guardian/next of kin.

## Author Contributions

All authors drafted the manuscript, and approved the final version of the manuscript. In addition, HA conducted the primary statistical analyses and ES was responsible for creating the figure.

### Conflict of Interest

The authors declare that the research was conducted in the absence of any commercial or financial relationships that could be construed as a potential conflict of interest.

## Footnotes

^1^www.moodprismapp.com
