# Supplementary material for: Mental Health Consequences of Adversity in Australia: National Bushfires Associated With Increased Depressive Symptoms, While COVID-19 Pandemic Associated With Increased Symptoms of Anxiety
Source: Front Psychol. 2021 May 19;12:635158. doi: 10.3389/fpsyg.2021.635158 (PMC8170463; doi:10.3389/fpsyg.2021.635158)
Supplement: Supplementary file 1 [file Table_1.docx]

**Supplement A**

Observed differences between time periods outlined in this study may be attributable to seasonal effects across time, such as the weather, time of year, or other annual events such as Christmas or holiday periods.

***Data Analysis plan***

To examine the above mentioned possibility, the study analyses were replicated using bushfire and COVID-19 equivalent time periods in the preceding year--01/09/2018 to 12/03/2019, and 13/03/2019 to 15/06/2019, respectively. An equivalent baseline period was not available as the earliest data collected in this study was limited to 02/11/2018. As such, supplementary comparisons are limited to utilising the bushfire and COVID-19 equivalent time periods.

An initial set of MLM analyses were conducted to confirm the effects of COVID-19 restrictions on mental health and wellbeing (rather than seasonal effects). This was achieved by conducting a MLM examining differences between the bushfire and COVID-19 equivalent periods in the preceding year.

Table 2.

*Partially standardised coefficients (standard errors) for individual MLMs comparing average outcome scores between the bushfire and COVID-19 equivalent periods in the preceding year*

| Outcome | Bushfires (2018/2019)† vs COVID-19 (2019) |
| --- | --- |
| Social connectedness | -0.05 (0.04) |
| Meaning and purpose | 0.04 (0.04) |
| Personal control | 0.10 (0.04)* |
| Arousal | 0.06 (0.05) |
| Sense of achievement | -0.01 (0.04) |
| Anxiety symptoms | 0.00 (0.04) |
| Motivation | 0.04 (0.04) |
| Self-esteem | -0.03 (0.04) |
| Emotional valence | 0.01 (0.05) |
| Depressive symptoms | 0.03 (0.04) |

*p < .05

** p < .01

***p < .001

† = comparator group

As shown in Table 2, with the exception of personal control, no significant differences were observed among any outcome when comparing the Bushfire and COVID-19 equivalent time periods in the preceding year.

A second set of MLM were conducted with direct comparisons between the bushfire and COVID-19 period with their respective equivalent time periods in the preceding year. The results of these analyses are shown in Table 3.

Table 3.

*Partially standardised coefficients (standard errors) for individual MLMs comparing average outcome scores between the (c) bushfire and (d) COVID-19 periods with their equivalent time periods in the preceding year.*

| Outcome | (c) Bushfires (2018/2019)† vs Bushfires (2019/2020) | (d) COVID-19 (2019)† vs COVID-19 (2020) |
| --- | --- | --- |
| Social connectedness | -0.10 (0.05) | -0.37 (0.05)*** |
| Meaning and purpose | -0.28 (0.05)*** | -0.40 (0.05)*** |
| Personal control | -0.04 (0.06) | -0.15 (0.06)** |
| Arousal | 0.04 (0.05) | -0.12 (0.05)** |
| Sense of achievement | -0.14 (0.06)** | -0.23 (0.05)*** |
| Anxiety symptoms | 0.01 (0.05) | 0.10 (0.05)* |
| Motivation | -0.08 (0.14) | -0.20 (0.05)*** |
| Self-esteem | -0.18 (0.05)*** | -0.23 (0.05)*** |
| Emotional valence | -0.09 (0.05) | -0.16 (0.05)*** |
| Depressive symptoms | 0.13 (0.05)* | 0.14 (0.05)*** |

*p < .05

** p < .01

***p < .001

† = comparator group

Several significant effects were observed in comparisons of the bushfire periods. These results showed that participants scored lower on items assessing meaning and purpose, sense of achievement, and self-esteem, and higher on the item assessing symptoms of depression. In analyses comparing the COVID-19 periods, results showed significantly poorer mental health and wellbeing across all outcome variables. This included significantly higher symptom scores on both anxiety and depression, and lower scores on all remaining variables.
